# Novel Aspects of CPAP Treatment and Interventions to Improve CPAP Adherence

**DOI:** 10.3390/jcm8122220

**Published:** 2019-12-16

**Authors:** Terri E. Weaver

**Affiliations:** Department of Biobehavioral Health Science, College of Nursing, University of Illinois at Chicago, Chicago, IL 60612, USA; teweaver@uic.edu; Tel.: +1-312-355-0118

**Keywords:** obstructive sleep apnea, CPAP, adherence, behavioral intervention, motivational enhancement therapy

## Abstract

Continuous positive airway pressure (CPAP) is an effective treatment for obstructive sleep apnea. However, the success of this treatment is hampered by nonadherence in half of the treated patients. Moreover, in clinical trials, poor adherence reduces adequate exposure required to determine its true effect. There is growing evidence that behavioral interventions, in addition to education, are a promising approach to improving adherence. Behavioral interventions include the use of cognitive behavioral therapy and motivational enhancement therapy designed to elevate a patient’s self-efficacy. The abundance of data obtained by CPAP tracking systems enables daily surveillance of use, and this telemonitoring along with telehealth allows the provider to quickly intervene when nightly CPAP use falls below thresholds or mask leaks are present. Telehealth reaches a large number of patients who may not be able to regularly attend a clinic, providing support and reinforcement. Peer support may also be useful in improving adherence. Not all obstructive sleep apnea patients present with the same phenotype, and can, therefore, be clustered into several groupings. Which intervention is most successful with a given phenotype or cluster remains unexplored. Comprehensive adherence management requires a team approach with the unique contribution of different professionals.

## 1. Introduction

Continuous positive airway pressure (CPAP) treatment for obstructive sleep apnea (OSA) improves daily functioning, cognitive processing, and mood, while positively affecting insulin resistance and possibly reducing the risk of stroke, hypertension, and cardiovascular disease [1]. The use of CPAP has also decreased traffic and other work-related accidents in addition to enhancing work productivity [1]. Despite these very significant effects that influence quality of life, adherence to this treatment remains a formidable obstacle to its effectiveness. Gains in both objective and subjective outcomes such as daytime sleepiness and daily functioning are greater with higher use, with the goal of >4 hours use per night [2,3,4]. Unfortunately, less than half of CPAP users apply it every night as prescribed [1]. Indeed, even skipping one night of treatment returns the number of apnea and hypopnea events, subjective and objective daytime sleepiness, and cognitive processing to baseline levels [5]. Not only does this limited treatment exposure affect clinical outcomes, it also limits the evaluation of the impact of CPAP on such comorbidities as cardiovascular disease, diabetes, hypertension, and cancer in clinical trials, underestimating the effect size. The purpose of this article is to present recent approaches to improving CPAP adherence that have shown promise. Models of care to more efficiently implement such interventions in the clinical setting will also be discussed.

## 2. One Size Does Not Fit All

Personalized care dictates that approaches to treatment will take into consideration differences in individual preferences and responses to treatment. Recently, there have been a number of studies that indicate phenotypes—clusters of OSA symptoms [6,7,8]. For example, the primary symptom of OSA, daytime sleepiness, does not manifest itself in some individuals [6]. Differences between those with objective and subjective daytime sleepiness, either objective or subjective sleepiness alone, or no sleepiness were related to education, race, sleep duration, morningness or eveningness tendencies, arousal index, and levels of TNFα [6]. Using cluster analysis, Ye and colleagues identified three distinct OSA clusters—disturbed sleep group (Cluster 1), minimally symptomatic group (Cluster 2), and excessive daytime sleepiness group (Cluster 3) [7]. Similarly, Gagnadoux and co-workers identified five clusters based on gender, presence of insomnia and comorbidities, depressive symptoms, and daytime sleepiness, as well as other typical nocturnal and diurnal OSA symptoms [8]. Device use lasting >4 hours/night and symptom improvement differed among the five clusters. Indeed, Cluster 1 (labeled female OSA), Cluster 4 (labeled mildly symptomatic OSA), and Cluster 5 (labeled comorbid OSA) had higher odds of using CPAP <4 hours/night than Cluster 3 (severe OSA syndrome). It seems logical that those patients experiencing greater symptoms would be more motivated to embrace treatment. Going beyond clinical criteria, this study provides additional prognostic information to alert the provider as to who may be of greater risk for nonadherence, thus requiring the investment of more time and alternative approaches to generate success. 

## 3. Methods to Enhance Self-Efficacy

### 3.1. Role of Self-Efficacy in Adherence

As the pattern of adherence is evident within the first week of CPAP treatment, it suggests that patients have already formed perceptions regarding the seriousness of OSA and the benefit of treatment, and thus these cognitions influence adherence. Based on this theoretical underpinning, the most effective approaches to promote adherence have been based on patient perception [9,10,11]. Applied to other situations of chronic illness, Bandura’s social cognitive theory posits that motivation is based on the perception of risk, appreciation of treatment benefit, and volition to engage in the behavior or self-efficacy [12,13]. Education is one way to enhance self-efficacy. It is an essential component of promoting adherence as recommended by the American Academy of Sleep Medicine [9]. Opportunities to provide educational content (see Box 1) to the patient include one-on-one clinic visits, follow-up telephone calls, telehealth interactions, and group meetings. In addition to the American Academy of Sleep Medicine clinical practice guidelines for the treatment of OSA [9], educational materials such as pamphlets and videotapes for both clinicians and patients are available from several internet sites (see the American Academy of Sleep Medicine: https://aasm.org; National Sleep Foundation: https://www.sleepfoundation.org/narcolepsy/homeward; American Sleep Apnea Association: https://www.sleepapnea.org/). It should be noted, however, that it has been shown that education alone has only a modest effect on CPAP application, i.e., increasing use by 35 minutes [14]. 

### 3.2. Behavioral Interventions

Applying social cognitive theory through methods associated with cognitive behavioral therapy (CBT) and motivational enhancement therapy (MET), behavioral interventions have shown considerable promise both in one-on-one and group sessions. Through conversational exchange, the goal of CBT is to correct the patient’s beliefs that are incorrect or unfounded in order to bring about a change in their behavior [15]. Motivational enhancement therapy applies motivational interviewing to elicit the patient’s thought processes, reinforcing the patient’s own motivating statements through directed interview questions [1,16]. Inherent in the application of MET is the assessment of the patient’s conviction and confidence in making a change [17]. Both MET and cognitive behavioral therapy can be delivered by either an advanced practice nurse or psychologist who has received training in these methods. 

The study by Richards and team demonstrated the effectiveness of cognitive behavioral therapy [11]. In this randomized controlled trial (RCT), participants were randomized to either usual care or group cognitive behavioral therapy of 10 participants/group. Usual care involved mask fitting and education. The experimental group watched a slide presentation and received a booklet on normal sleep, health consequences of OSA, and effectiveness of CPAP treatment, as well as viewing, but not handling a CPAP machine. CBT was delivered in two 1-hour CBT sessions in addition to relaxation techniques. After 28 days, those who received CBT were more likely to take the device home, had a mean daily CPAP use of 5.38 hours use/night versus 2.51 hours use/night when compared with usual treatment, and had more nights with use >4 hours/night. Those in the experimental arm also had greater self-efficacy. These outcomes are consistent with the theoretical underpinnings of CBT that greater self-efficacy is associated with higher levels of adherence [18].

The majority of studies employing a behavioral intervention to improve CPAP have used MET [16]. For example, Bakker et al. performed a secondary analysis of data from the RCT BestAir study that used MET to promote adherence, in the investigation of CPAP on cardiovascular comorbidity in OSA [19]. They examined adherence at 12 months, comparing the experimental MET group and those receiving usual care. In-person 1-hour MET sessions were delivered by a trained psychologist at baseline and week 1, followed by 30-minute phone calls by the psychologist at weeks 3, 4, 8, 12, 20, and 32. The MET group on average had better adherence outcomes, which were statistically reliable, using CPAP 80 minutes longer per night than the usual care group. Olsen et al., in their application of MET, used three sessions; the first two were 30–45 minutes long and the final session was 20–30 minutes [20]. The content of the first session was designed to build motivation for change and elicit the participant’s motivation to treat their sleep problem, discussed the importance of treatment and CPAP as the prime therapeutic choice. The aim of the second session was to strengthen the commitment to change, where the pros and cons of receiving treatment and jeopardies regarding not using CPAP were discussed. The final session was a booster session to reinforce change and underscore the goals for CPAP use. CPAP acceptance was defined in the study as the percentage of participants who started CPAP within three months. The rejection rate of CPAP was 6% for the MET group compared to 28% for the control group and remained statistically significant at the 12-month follow-up. Differences in nightly CPAP use between the two groups were present for each of the first three months, but not after 1 year. However, when considering all participants who used CPAP by three months, there were no statistically significant differences at any of the time points postinitiation, which the authors interpreted as an indication that MET has more impact on acceptance of CPAP than adherence rates. Of note was the increase in self-efficacy in the MET arm.

A Cochran Review has assessed that behavioral therapy produces a considerable improvement in nightly use of just under 1.5 hours, along with a greater proportion of patients who used their devices for more than 4 hours/night [14]. What remains to be determined is which behavioral intervention, such as CBT or MET, is best to promote optimum success for the different patient phenotypes [14]. Due to the limited number of studies that have examined the cost of behavioral intervention relative to staff time and associated benefit, generating cost/benefit data is critical to the utilization of all forms of behavioral therapy.

### 3.3. Telemonitoring/Telehealth—Data Access and Distance Communication

A novel aspect of CPAP devices is the machine or web-based tracking systems that generate information of high utility to both the provider and the patient. Information on nightly as well as longer periods of CPAP use can be obtained and utilized to evaluate the effectiveness of treatment and level of adherence. These applications indicate the presence of mask leaks and residual sleep-disordered breathing, and nightly CPAP duration over designated periods of time, as well as various flow signals [21]. Some systems include coaching and troubleshooting information in addition to education. As illustrated by the algorithm developed by Schwab and co-authors, these data contribute to early troubleshooting and decision-making in care management [21].

A limited number of studies have investigated the impact of telemonitoring on CPAP adherence [16]. Unfortunately, they have produced conflicting results [14,16,22]. For example, in a single-center RCT comparing telemonitoring to usual care in participants with moderate to severe OSA, adherence data were sent via a modem and reviewed daily by research staff [23]. Participants were contacted to troubleshoot issues if there was a leak, residual events, or less than four hours use on two consecutive days. If the only problem was adherence, the research coordinator provided encouragement to use CPAP. Other issues were addressed with clinic staff. After 4–6 weeks, the participant returned to the clinic and data from the tracking system were reviewed. After 3 months of treatment, the participant returned and downloaded data were reviewed by the physician. Compared to usual care, there were significant differences in mean minutes of use per day and mean minutes of use on nights used, with an increase of almost 2 hours on nights used. Also, using specific metrics for intervention, Frasnelli and coworkers employed guidelines for interventions when nightly use was <4 hours/night for two days, or if a leak of >0.5 l/sec was present for more than two days, as indicated in data downloaded three days per week [24]. After 30 days of use, telemonitoring was superior to usual care in hours of use per night, number of days used, nights with >4 hours use, and use on ≥70% of the nights. 

In another RCT, 100 participants were assigned to either usual care or telemonitoring over 3 months [25]. In this study by Turino and colleagues, metrics for thresholds and alerts were employed similar to that of Fox et al. [23,25]. An alarm was generated when thresholds were reached, and the physician contacted the participant and engaged in problem-solving and support for the use of CPAP. At one month, nightly use for the standard care group was 5.2 ± 2.1 hours/night compared to the telehealth group where use was 4.8 ± 2.3 hours/night, which was not statistically significant. Similar results (4.9 ± 2.2 hours/night, 5.1 ± 2.1 hours/night, respectively) were found at three months. However, telehealth costed less (EUR123.65 versus EUR170.97; *p* = 0.022) and was more cost-effective in financial investment relative to quality-adjusted life-years gained.

Telehealth was developed to provide health care services at a distance and can be used for assessment, diagnosis, treatment, obtaining/retrieving data, and evaluation [16]. Moving beyond education, there is evidence that providing feedback through CPAP tracking systems, web sites, and apps may alter perception and produce greater gains. Studies have also employed telehealth to provide automatic feedback to improve CPAP adherence [16,22]. Using data transferred via wireless modem, Munafo and team members considered differences in adherence between participants randomized to usual care versus those receiving email messages with follow-up on days 1, 7, 14, 30, and 90 [26]. The telehealth group received the same education as the control, but only received a phone call on day 90, unless it was in response to an automated message regarding greater than one of five key parameters: median mask leak >24 L/min for 2 consecutive days; no CPAP data for two consecutive days; CPAP use <4 hours for three consecutive nights; residual apnea-hypopnea index (AHI) >15/hour for five consecutive days; CPAP usage met Medicare criteria for adherence (>4 hours CPAP usage each night for 70% of nights during a 30 consecutive-day period anytime during the first 90 days of initial usage). In the intent to treat 138 participants, there were no differences between groups in Medicare adherence rates. 

Whether telehealth is more effective than telemonitoring remains uncertain. Are these interventions superior to stand-alone education, or does a combination of all provide better adherence outcomes? A large study by Hwang and colleagues was designed to answer these questions, employing a four-arm RCT—two telehealth interventions (web-based OSA education [Tel-Ed] and CPAP telemonitoring program with automated feedback messaging [Tel-TM]), a combination of the two, and usual care [27]. Before treatment was initiated, the Tel-Ed group was emailed a link to a program that provided education about OSA, health-related risks, comorbidities, the risks of daytime sleepiness, and information about the assessment process. In the first week of treatment, a second link was provided, which had instructions on the use and care of the CPAP device and expected outcomes. The Tel-TM group was oriented with an automated feedback process and was asked their preference for receiving text messages, emails, phone calls, or a combination. If CPAP use met the adherence criteria, a message of positive reinforcement was provided. Of the Tel-TM and combination groups, the majority preferred to receive messages via email (31.7%), followed by text messaging (17.5%), and phone (2.3%). Forty percent preferred a combination of approaches. There were no differences between the Tel-Ed group and the other treatment arms for percent of days used, average use on all days, and average use on the days CPAP was used. However, there were statistically significant differences in these outcomes for the Tel-TM group compared with the combination of Tel-Ed and Tel-TM. Of particular interest is the finding that due to a protocol violation, the Tel-TM messaging continued after the 90-day cutoff period. Although the other groups had a decline in use, this group did not, possibly due to the continued reinforcement. This investigation helps us better understand the relative contribution to telehealth and telemonitoring and demonstrates enhanced use with a system that requires limited provider intervention. It must be recognized that access to the required technology, both from the provider and patient standpoint, is a limitation to widespread use. This impedance will likely diminish over time as the availability of technology increases and cost decreases. The success of telehealth/monitoring may also be dependent on knowing who best responds (age, education, geographic setting) to the different telehealth/monitoring approaches. 

## 4. Comprehensive Adherence Management

Success in promoting CPAP adherence is dependent on the utilization of the health care team to bring each discipline’s perspective to bear on problem-solving. Effective teams consist of the physician, technologist, advanced practice nurse, psychologist, and importantly, the partner or other resource with whom the patient has formed a relationship. Interviewing 20 couples, Ye and colleagues found that the following enhanced adherence [28]:

- Having the partner contribute to the diagnosis and treatment
- The partner and patient being jointly engaged in coping and problem-solving CPAP issues
- Both the patient *and* the partner perceived benefit of treatment
- The patient was motivated to use CPAP on behalf of the partner
- Overall support provided by the partner

Gentina found that the quality of the marriage and engagement of the partner affected nightly CPAP use [29]. Thus, having the partner involved as a member of the team is essential, where a positive relationship exists, to producing positive adherence outcomes. The influence of someone who can relate to the patient may extend beyond the partner. In a Veterans Administration facility, Parthasarathy recruited successful CPAP users to serve as buddies for patients newly diagnosed with OSA [30]. Buddies were trained but did not give medical advice. The support and frequent interaction of the buddies resulted in greater satisfaction with the program and greater CPAP use after 90 days of treatment when compared to usual care. Patient satisfaction was positively related to the level of CPAP adherence.

As machine use is determined within the first week of treatment, it is likely that patients approach CPAP therapy with opinions regarding the diagnosis and value of treatment before therapy is initiated. Thus, changing these views takes considerable effort, but is well within the boundaries of clinical practice if a chronic disease management approach is utilized. Indeed, hub-and-spoke models have been proposed that depict the sleep center as the “hub” with primary care, delivered by physicians or advanced practice nurses, and include telehealth locations as the “spokes” [31]. This model increases the resources available beyond just the sleep center to monitor and introduce early adherence with the sharing of tracking system data and outcomes of intervention.

## 5. Conclusions

Adherence to CPAP treatment for OSA is essential to achieve the highest potential quality of life and cognitive processing outcomes, decrease accidents, and reduce comorbidities. Unlike other treatments for chronic disease, the CPAP device offers quality data that provides information on hour-to-hour, daily, and monthly use, along with deterrents to use such as the presence of leaks or residual events. Despite having these data, adherence levels remain unchanged, with approximately half of patients being nonadherent [32]. When directed towards altering patient views on CPAP, behavioral intervention appears to be the most effective intervention to promote adherence. Telemonitoring enables early intervention with alerts to complications or changes in use. Telehealth, like behavioral approaches, creates additional opportunities to interface with a provider, delivering greater support, education, and intervention. Unexplored is the value of a combination of these approaches and unclear is which patient phenotype, or cluster, best responds to which approach. Although models of care delivery to enhance CPAP use have been presented, further investigation regarding their utility, implementation, and cost analysis is needed. 

As a final note, providers should be aware that about 20% of patients who are highly adherent to treatment remain sleepy. Mechanisms for this have been proposed, and there is evidence of white matter compromise, with myelin diffusivity affected along with axonal shrinkage, in those who are sleepy compared with adherent patients who are not [33]. Fortunately, in these cases, there are medications that increase alertness with better sustained attention and quality of life [34,35]. These include modafinil and solriamfetol. Causes for sustained sleepiness should be evaluated, and when no apparent factor is identified, these agents should be prescribed so that patients do not become frustrated with sustained symptomatology and abandon treatment.

## Supplementary Materials

The following are available online at https://www.mdpi.com/2077-0383/8/12/2220/s1, Box 1: Patient Education—OSA and CPAP Treatment.
