# Supplementary material for: Novel Aspects of CPAP Treatment and Interventions to Improve CPAP Adherence
Source: J Clin Med. 2019 Dec 16;8(12):2220. doi: 10.3390/jcm8122220 (PMC6947399; doi:10.3390/jcm8122220)
Supplement: Supplementary file 1 [file jcm-08-02220-s001.pdf]

Box 1

Patient Education - OSA and CPAP Treatment

- Sleep Hygiene
  - Regular schedule, especially rise time
  - Avoid stimulants, alcohol, caffeine, nicotine
  - Set aside a worry time
  - Keep sleeping room cool, dark, quiet
  - Use bed only for sleep and sex
  - Limit naps to 30 min
  - Avoid large meals; or emotional activities prior to retiring to bed
  - Upon awakening, get up out of the bed, no matter what the time is
  - Avoid looking at the clock - turn the clock around
- Pathophysiology
- Risk Factors and their modification
- Consequences of OSA
- Weight loss
- CPAP therapy
  - CPAP as pneumatic splint to prevent apneas and hypopneas
  - Outcomes of CPAP treatment including decreased sleepiness/greater alertness, comorbidity risk reduction, improved mood, better quality of life, etc.
  - Importance of nightly use for entire sleep duration for greatest benefits
  - Equipment and device selection, especially mask options (some of this may be covered by the sleep laboratory)
  - Handling of side effects and troubleshooting potential problems
- Positional therapy for those with apneas and hypopneas predominantly in supine position
- Hypoglossal nerve stimulation and other treatment options including pharmaceutical agents
